# The mechanism of lncRNAs in the crosstalk between epithelial-mesenchymal transition and tumor microenvironment for early colon adenocarcinoma based on molecular subtyping

**DOI:** 10.3389/fgene.2022.997739

**Published:** 2022-11-16

**Authors:** Hanlin Liang, Yi Zhao, Kai Liu, Yajie Xiao, Kexu Chen, Delan Li, Shupeng Zhong, Zhikun Zhao, Dongfang Wu, Yu Peng

**Affiliations:** ^1^ Chemotherapy Department, Zhongshan City People’s Hospital, Zhongshan, China; ^2^ GI Medicine, The Third Hospital Affiliated to Naval Medical University, Shanghai, China; ^3^ Department of Colorectal Oncology, National Clinical Research Center for Cancer, Key Laboratory of Cancer Prevention and Therapy of Tianjin, Tianjin’s Clinical Research Center for Cancer, Tianjin Medical University Cancer Institute and Hospital, Tianjin, China; ^4^ Department of Medicine, YuceBio Technology Co., Ltd., Shenzhen, China; ^5^ Oncology Department, Jiangmen Central Hospital, Jiangmen, China

**Keywords:** colon adenocarcinoma, epithelial-mesenchymal transition, long non-coding RNAs, tumor microenvironment, transcription factors, biomarkers, bioinformatics analysis

## Abstract

A large number of colon adenocarcinoma (COAD) patients are already advanced when diagnosed. In this study, we aimed to further understand the mechanism of tumor development in early COAD by focusing on epithelial-mesenchymal transition (EMT) and long non-coding RNAs (lncRNAs). Expression profiles of early COAD patients were obtained from public databases. EMT-related lncRNAs were used as a basis for constructing molecular subtypes through unsupervised consensus clustering. Genomic features, pathways and tumor microenvironment (TME) were compared between two subtypes. LncATLAS database was applied to analyze the relation between lncRNAs and transcription factors (TFs). First order partial correlation analysis was conducted to identify key EMT-related lncRNAs.C1 and C2 subtypes with distinct prognosis were constructed. Oncogenic pathways such as EMT, KRAS signaling, JAK-STAT signaling, and TGF-β signaling were significantly enriched in C2 subtype. Higher immune infiltration and expression of immune checkpoints were also observed in C2 subtype, suggesting the key EMT-related lncRNAs may play a critical role in the modulation of TME. In addition, JAK-STAT signaling pathway was obviously enriched in upregulated TFs in C2 subtype, which indicated a link between key lncRNAs and JAK-STAT signaling that may regulate TME. The study further expanded the research on the role of EMT-related lncRNAs in the early COAD. The six identified EMT-related lncRNAs could serve as biomarkers for early screening COAD.

## Introduction

According to the global cancer statistics, colon adenocarcinoma (COAD) is the sixth most diagnosed cancer with 1,148,515 new cases in 2020, contributing 6.0% of all new diagnosed cancer cases (Sung et al., 2021). Simultaneously, the death cases contribute 5.8% (576,858) of all deaths by cancer, which is the fifth leading cause of cancer death in 2020. Although the 5-year overall survival (OS) is upon 75% of American Joint Committee on Cancer (AJCC) stage Ⅰ and Ⅱ, a dramatically decreased survival of distant metastasis is shown with only less than 20% of AJCC stage Ⅳ (Ulanja et al., 2019). Prognostic difference is shown between right-sided and left-sided colon cancer, and left-sided colon cancer has a lower death risk than right-sided colon cancer (Petrelli et al., 2017), which may result from their genetic and immunological differences (Lee et al., 2015). Screening techniques for colon cancer include invasive and non-invasive tests. Colonoscopic tests are recommended for high-risk individuals and non-colonoscopic tests are recommended in average-risk individuals according to European Society for Medical Oncology (EMSO) clinical practice guidelines (Argilés et al., 2020). However, they are not sensitive in the diagnosis of early colon cancer. Actually, a number of patients are already advanced when diagnosed as COAD. Therefore, early screening of COAD is of great value for improving prognosis and releasing cancer burden worldwide.

To reach accurate screening, comprehensive understanding of COAD tumorigenesis and development is a basis for identifying effective biomarkers for COAD screening. Of the hallmarks of cancers, epithelial-mesenchymal transition (EMT) is one of the most important features contributing for metastasis (Dongre and Weinberg, 2019). Tumor microenvironment (TME), another important component in cancer tissue, has been demonstrated to have a strong correlation with EMT through the linkages of proinflammatory factors such as TGF-β, TNF-α, and IL-6 (Jung et al., 2015). Among these interactions, long non-coding RNAs (lncRNAs) are considered as critical regulators for modulating TME and managing EMT (Sun et al., 2018; O'Brien et al., 2020). Oncogenic pathways involving in EMT such as WNT signaling, JAK-STAT3 signaling (Xue et al., 2018), mTOR signaling and MAPK/ERK signaling have been illustrated to be regulated by various lncRNAs (O'Brien et al., 2020). In colon cancer, lncRNA-HOTAIR associated with EMT was identified as a predictor of metastasis and prognosis (Wu et al., 2014).

As lncRNAs are of potential to serve as biomarkers for COAD prognosis, we consider that lncRNAs involving in EMT process may also be effective predictors for COAD. Therefore, in this study, we tried to construct a novel molecular subtyping system based on EMT-related lncRNAs. Compared to pathological subtyping or clinical features, molecular subtyping is more accurate for classifying cancer patients into different classes with different prognosis. By exploring the mechanisms of EMT-related lncRNAs in tumorigenesis in COAD, we further identified six key EMT-related lncRNAs that could serve as biomarkers for early screening COAD.

## Materials and methods

### Sample collection

COAD samples were obtained from public databases through Sangerbox platform (Shen et al., 2022). RNA-seq data with clinical information was downloaded from The Cancer Genome Atlas (TCGA) database. GSE17538 with gene expression profiles was downloaded from Gene Expression Omnibus (GEO) database. In TCGA-COAD and GSE17538 cohorts, only samples of stage I and II were retained, and samples without survival information were removed. The clinical information of COAD samples was shown in Table 1.

**TABLE 1:** The clinical information of COAD samples.

Clinical features	TCGA-COAD	GSE17538
OS		
0 (alive)	199	80
1 (dead)	26	20
T Stage		
T1	8	
T2	61	
T3	145	
T4	10	
TX	1	
N Stage		
N0	225	
M Stage		
M0	204	
MX	21	
Stage		
I	70	28
II	155	72
Gender		
Female	99	47
Male	126	53
Age		
<=70	117	53
>70	108	47

### Identification of epithelial-mesenchymal transition-related lncRNAs

EMT-related genes in hallmark EMT pathway were obtained from Molecular Signatures Database (MSigDB, v7.4, https://www.gsea-msigdb.org/gsea/msigdb/) (Liberzon et al., 2015). LncRNAs and mRNAs in TCGA-COAD and GSE17538 cohorts were annotated by gene transfer format (GTF, v32) file which was downloaded from GENCODE (https://www.gencodegenes.org/).

EMT score of each sample was calculated by single sample gene set enrichment analysis (ssGSEA) in GSVA R package (Hänzelmann et al., 2013). Then Pearson correlation analysis was employed to calculate correlation coefficients between EMT score and expression of lncRNAs. EMT-related lncRNAs were determined by the conditions of |coefficient| > 0.25 and *p* < 0.05.

### Identification of molecular subtypes based on epithelial-mesenchymal transition-related lncRNAs

Screened EMT-related lncRNAs that were overlapped in TCGA-COAD and GSE17538 cohorts were used as a basis to construct molecular subtypes. Unsupervised consensus clustering in ConsensusClusterPlus R package was implemented to construct consensus matrix (Wilkerson and Hayes, 2010). KM algorithm and Euclidean distance were used to conduct 500 bootstraps with each bootstrap containing 80% samples. Cluster number k from 2 to 10 was included to screen the optimal cluster according to consensus matrix and cumulative distribution function (CDF).

### Gene set enrichment analysis

GSEA is a powerful analytical method for interpreting biological processes based on gene expression profiles (Subramanian et al., 2005), which was applied to assess hallmark pathways for two subtypes. Hallmark pathways with a series of gene sets were obtained from MSigDB (Liberzon et al., 2015). The proportion of 28 immune cells was estimated by GSEA based on gene signatures of different cell types (Şenbabaoğlu et al., 2016). Estimation of STromal and Immune cells in MAlignant Tumours using Expression data (ESTIMATE) (Yoshihara et al., 2013), also based on GSEA was used to calculate stromal score and immune score of two subtypes. ClusterProfiler R package was used to annotate Kyoto Encyclopedia of Genes and Genomes (KEGG) pathways on TFs and EMT-related lncRNAs (Yu et al., 2012).

### Localization of lncRNAs and calculation of transcription factor activity

Relative concentration index (RCI) based on LncATLAS database was introduced to measure the localization of lncRNAs (Mas-Ponte et al., 2017). RCI >0 indicates lncRNAs localizing in the cytoplasm and RCI <0 indicates the nuclear. The TF activity was assessed according to the algorithm from Garcia-Alonso et al. Garcia-Alonso et al. (2018). Pearson correlation analysis was conducted to analyze the association between EMT-related lncRNAs and TFs.

### Identification of key epithelial-mesenchymal transition-related lncRNAs

First order partial correlation analysis was used to evaluate the linkage among EMT-related lncRNAs, EMT score and EMT-related genes (Reverter and Chan, 2008). The association between two variables were largely decreased when eliminating the effect of another variable, and the variable was considered as key EMT-related lncRNA strongly associated with EMT score and EMT-related genes.

The identified key EMT-related lncRNAs were used to construct a prognostic model for predicting OS. Univariate Cox regression analysis was conducted on these lncRNAs and coefficients were generated for building the model, defining as: risk score =Σ (beta i × exp i). Beta represents coefficients and exp represents the expression of lncRNAs.

### Statistical analysis

Statistical analysis was performed in R (4.1.1) software. Parameters of R packages and software were default if no introduce. Statistical methods were indicated in the corresponding figure legends. *p* < 0.05 was considered as significant. ns, no sifnificance. *p < 0.05, **p < 0.01, ***p < 0.001, ****p < 0.0001.

## Results

### Constructing two molecular subtypes based on epithelial-mesenchymal transition-related lncRNAs

**FIGURE 1:**
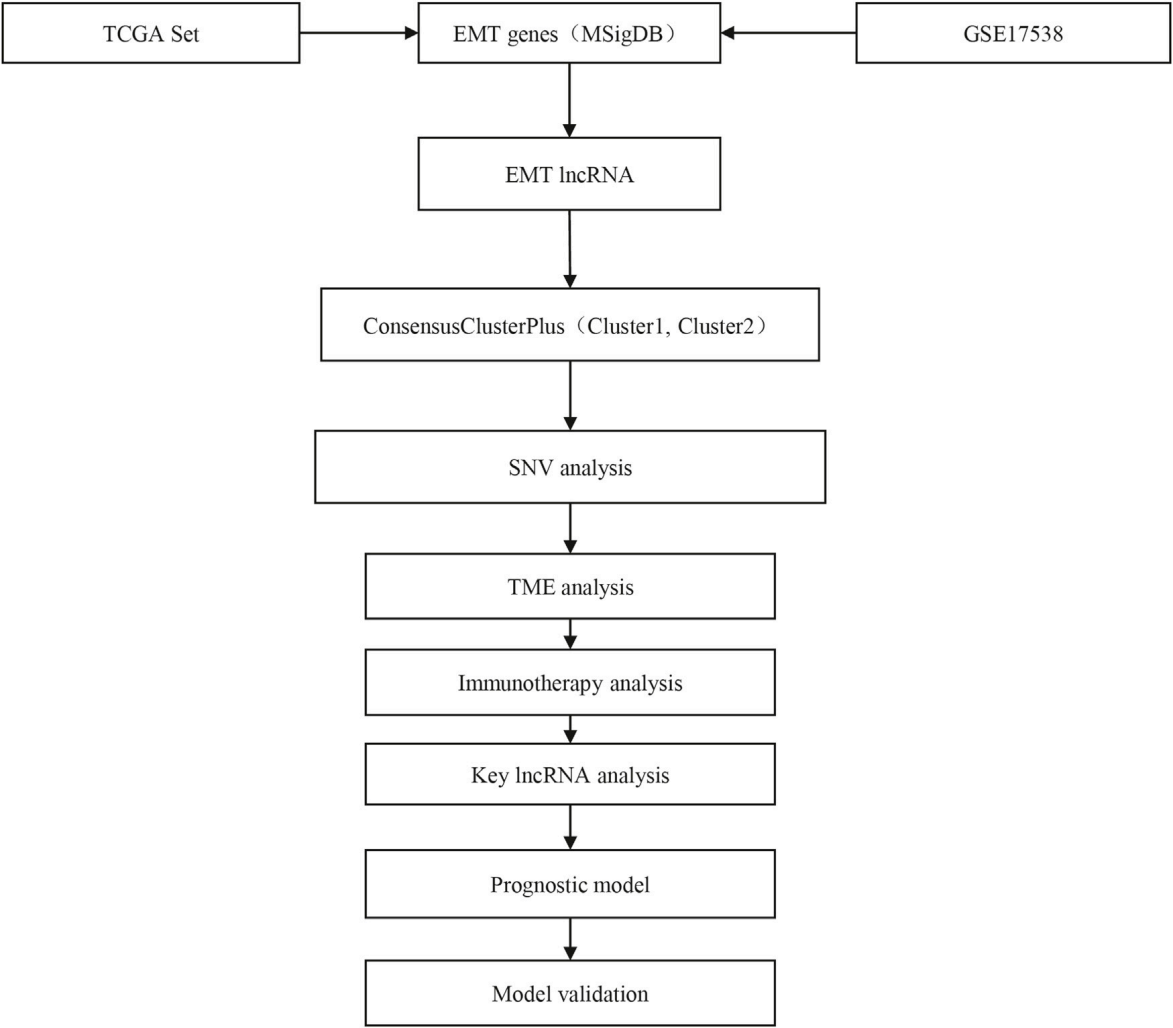
The work flow of this study.

**FIGURE 2:**
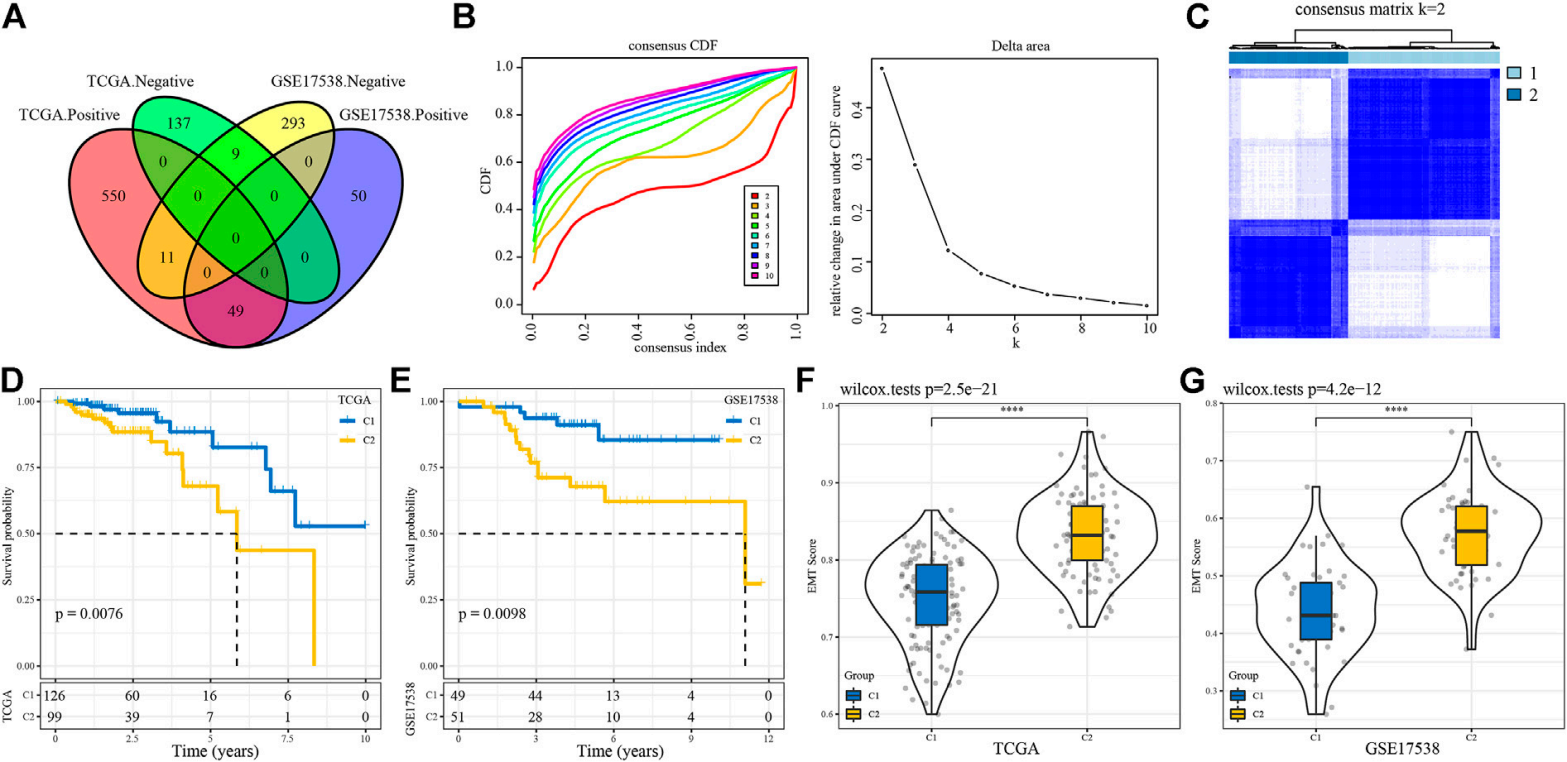
Construction of molecular subtypes based on EMT-related lncRNAs **(A)** Venn plot of lncRNAs positively or negatively correlated with EMT activity. **(B)** CDF curves of different cluster numbers (k) from 2 to 10 **(C)** Consensus matrix when cluster number k = 2. **(D–E)** Kaplan-Meier survival curves of C1 and C2 subtypes in TCGA-COAD **(D)** and GSE17538 **(E)** cohorts. Log-rank test was conducted. **(F–G)** Differential EMT score of C1 and C2 subtypes in two cohorts. Wilcoxon test was conducted. ****p < 0.0001.

The work flow of this study was shown in Figure 1. To identify EMT-related lncRNAs, Pearson correlation analysis was conducted between EMT activity and lncRNA expression. A total of 756 and 412 EMT-related lncRNAs were identified in TCGA-COAD and GSE17538 cohorts respectively (Figure 2A). Then the intersected part of 58 EMT-related lncRNAs were used as a basis for unsupervised consensus clustering. The optimal cluster number (k) was determined according to CDF curve and consensus matrix (Figures 2B,C). When k = 2, samples were obviously divided into two groups. Kaplan-Meier survival analysis of two groups showed a significance of OS in both two cohorts (*p* = 0.0076 and *p* = 0.0098 in TCGA-COAD and GSE17538 respectively, Figures 2D,E). Finally, COAD samples were classified into two molecular subtypes (C1 and C2), with C1 subtype had a superior OS than C2 subtype. EMT activity shown as EMT score also varied largely between two subtypes. Not surprisingly, C2 subtype exhibited a significantly higher EMT score than C1 subtype, indicating that EMT pathway was more activated in C2 subtype (*p* < 0.0001, Figures 2F,G). In addition, the result also demonstrated that these 58 EMT-related lncRNAs may play a key role in regulating EMT activity.

### Characterizing gene mutations of two molecular subtypes

**FIGURE 3:**
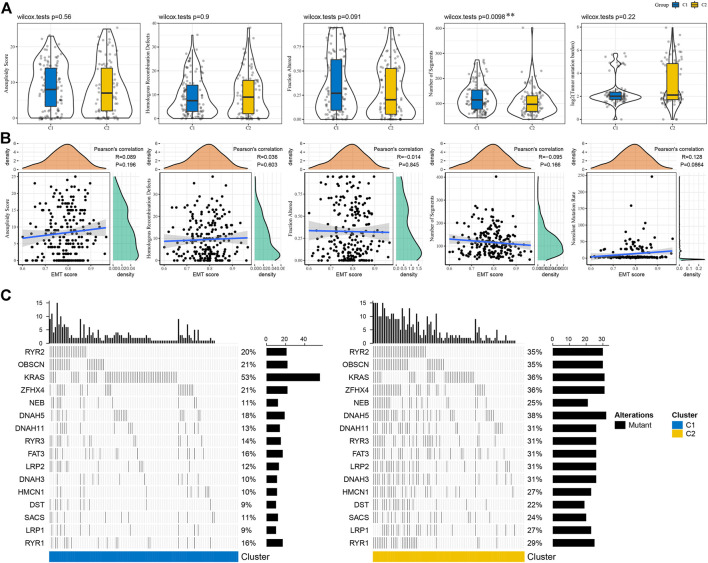
Mutation patterns of C1 and C2 subtypes **(A)** Genomic features of two subtypes including aneuploidy, homologous recombination defects, fraction altered, number of segments and tumor mutation burden. Wilcoxon test was performed. **(B)** Pearson correlation analysis between genomic features and EMT score. **(C)** The top 20 significantly mutated genes in C1 and C2 subtypes. Fisher exact test was conducted. **p < 0.01.

We compared the genomic features between C1 and C2 subtypes in TCGA-COAD cohort on five aspects including aneuploidy, fraction altered, tumor mutation burden, homologous recombination defects and number of segments. We observed that only a significant difference was shown in number of segments between two subtypes (Figure 3A). No obvious correlation was manifested between genomic features and EMT score (Figure 3B). Gene mutation analysis revealed that samples in C2 subtype had a higher mutated proportion than C1 subtype, except for *KRAS* mutations contributing for 53% samples in C1 subtype (Figure 3C).

### Oncogenic pathways were more enriched in C2 subtype

**FIGURE 4:**
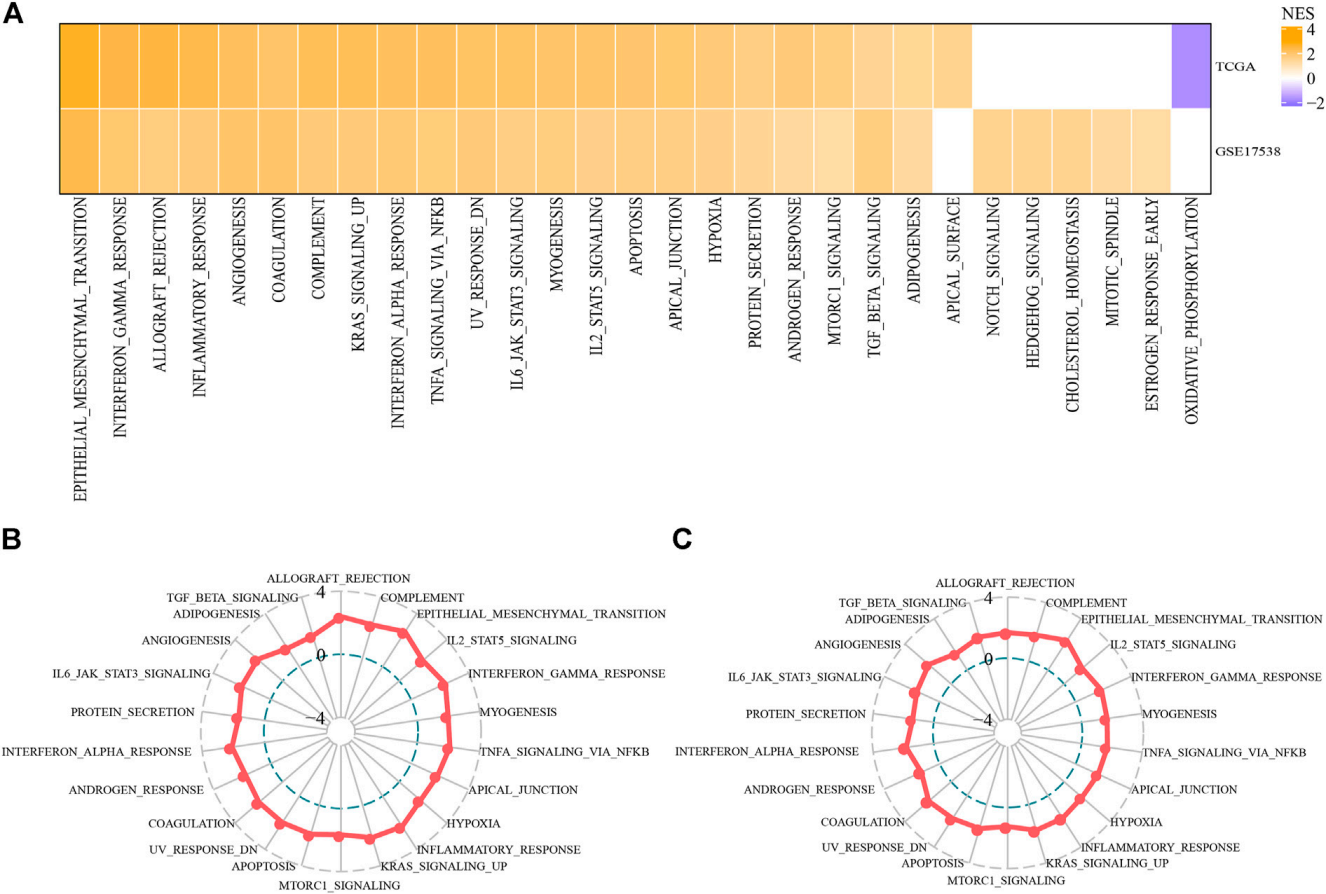
Differentially enriched pathways between C1 and C2 subtypes **(A)** Activated hallmark pathways by comparing C2 with C1 based on normalized enrichment score (NES). Orange indicates upregulated pathways in C2 and purple indicates the reverse **(B–C)** Rader plots of activated pathways of C2 subtype in TCGA-COAD **(B)** and GSE17538 **(C)** cohorts. NES was indicated as −4, 0 and 4 from inside to outside. Hallmark pathways were shown around rader plots.

Next we tried to know if there was a difference of activated pathways between two subtypes. Hallmark pathways from MSigDB were included for GSEA and significantly enriched pathways were outpuuted with false discovery rate (FDR) < 0.05. By comparing C2 subtype with C1 subtype, we observed that C2 subtype had 23 activated and 11 suppressed pathways in TCGA-COAD cohort, and 27 activated and 9 suppressed pathways in GSE17538 cohort. Of these activated pathways, we found that oncogenic pathways and immune-related pathways were greatly enriched, such as EMT, angiogenesis, KRAS signaling, hypoxia, interferon response, TNF-α signaling, IL6-JAK-STAT3 signaling, IL2-STAT5 signaling, and TGF-β signaling pathways (Figure 4A). Overall, C2 subtype had significantly higher normalized enrichment scores (NES) of these pathways than C1 in both two cohorts (Figures 4B,C), suggesting that EMT-related lncRNAs may be extensively involved in the regulation of these pathways especially EMT.

### C2 subtype had higher infiltration of immune cells

**FIGURE 5:**
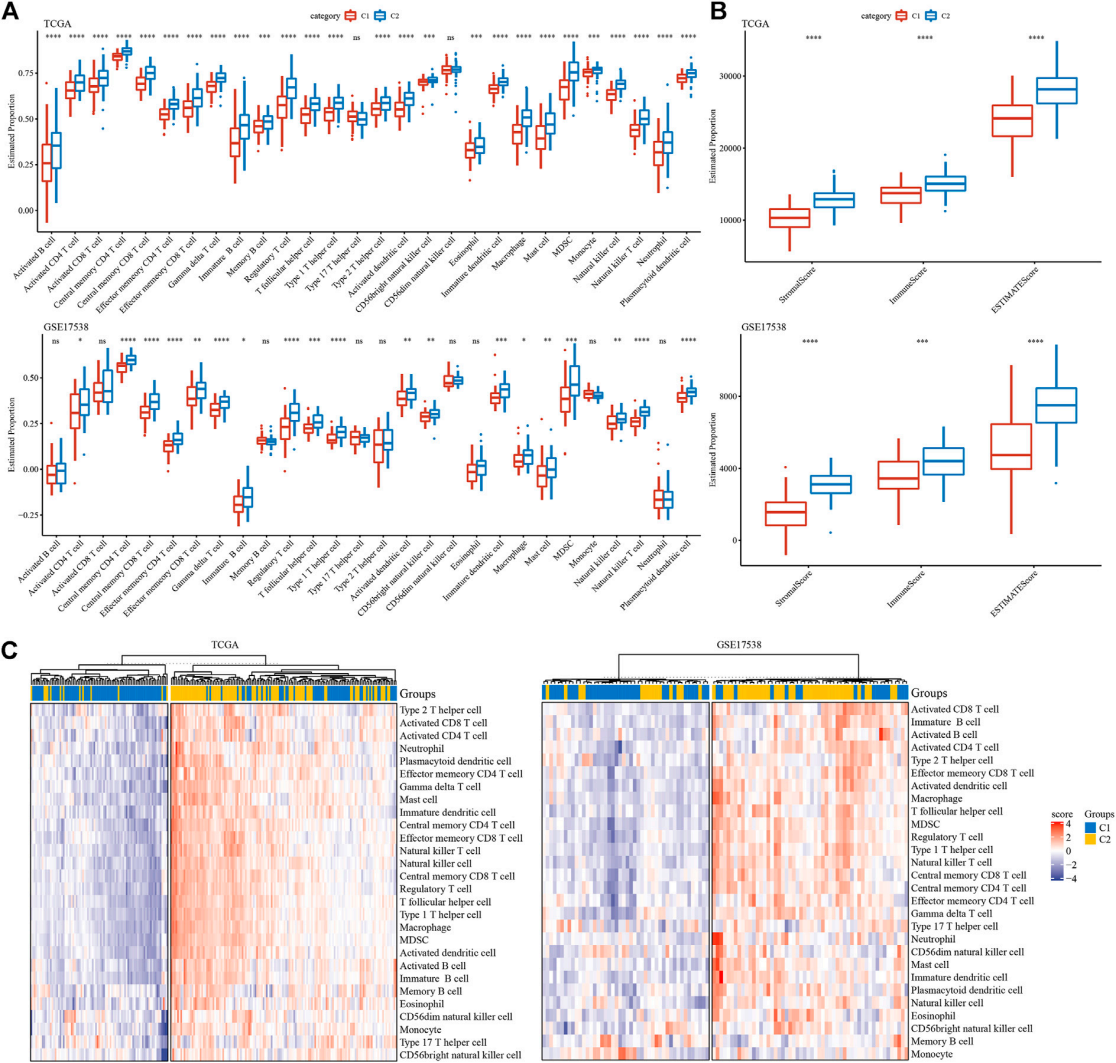
Tumor microenvironment of C1 and C2 subtypes **(A)** Estimated proportion of 28 immune cells in TCGA-COAD and GSE17538 cohorts. **(B)** Stromal score and immune score in TCGA-COAD and GSE17538 cohorts calculated by ESTIMATE. **(C)** Unsupervised consensus clustering based on gene signatures of immune cells in two cohorts. Red and purple indicates relatively high and low enrichment. Student *t* test was conducted between two groups. ns, no significance. *p < 0.05, **p < 0.01, ***p < 0.001, ****p < 0.0001.

To evaluate the tumor microenvironment of C1 and C2 subtypes, we assessed the estimated proportions of a series immune cells based on gene signatures from Şenbabaoğlu et al. Şenbabaoğlu et al. (2016). To our surprise, C2 subtype had extremely higher proportions of most immune cells in both two cohorts (Figure 5A). Specifically, activated CD4 T cells, activated CD8 T cells, regulatory T cells, dendritic cells, macrophages, myeloid-derived suppressor cells (MDSCs) and natural killer cells were all more enriched in C2 subtype. ESTIMATE evaluation also supported the result that C2 subtype had higher stromal score and immune score than C1 subtype in both two cohorts (*p* < 0.001, Figure 5B). Furthermore, unsupervised consensus clustering based on these immune cells clearly divided samples into two groups of high and low immune infiltration (Figure 5C). Obviously, samples in high immune infiltration group largely belonged to C2 subtype. These results implicated a close link between EMT-related lncRNAs and TME modulation.

**FIGURE 6:**
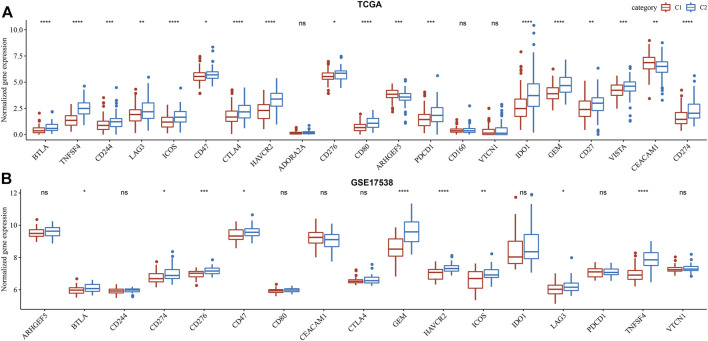
Comparison of immune checkpoint expression between two subtypes in TCGA-COAD **(A)** and GSE17538 **(B)** cohorts. Student *t* test was conducted. ns, no significance. *p < 0.05, **p < 0.01, ***p < 0.001, ****p < 0.0001.

Commonly, high immune infiltration of cytotoxic immune cells has favorable prognosis. However, immunosuppressive immune cells such as regulatory T cells and MDSCs were simultaneously increased in C2 subtype. In addition, we analyzed the expression of immune checkpoints obtained from HisgAtlas database (Liu et al., 2017). Higher expression of many important immune checkpoints was observed in C2 subtype, such as LAG3, ICOS, CTLA4, CD276, PDCD1, IDO1 and CD274 (Figure 6).

### The crosstalk between epithelial-mesenchymal transition-related lncRNAs and transcription factors

**FIGURE 7:**
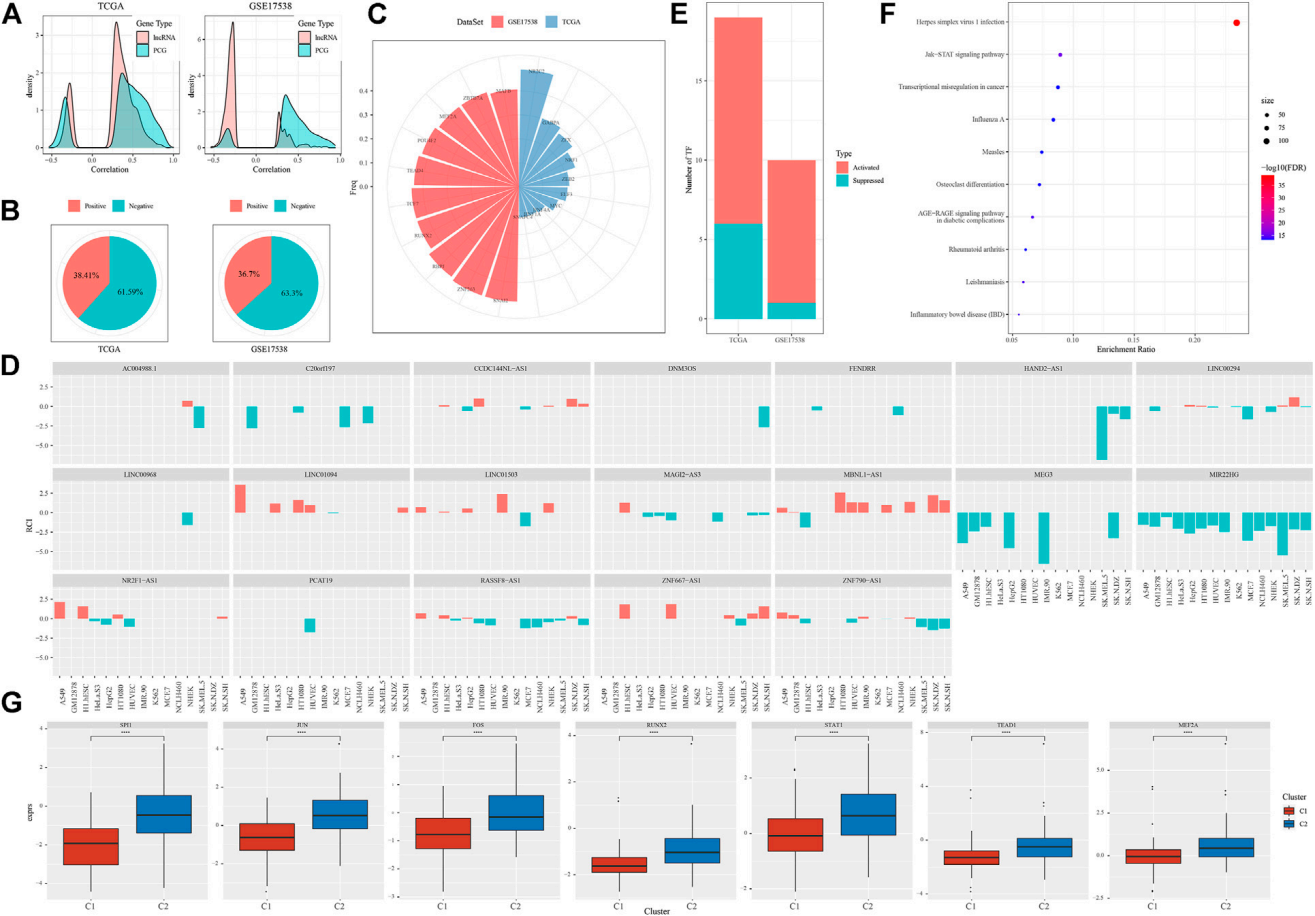
The relation between EMT-related lncRNAs and TFs **(A)** Pearson correlation analysis between EMT-related lncRNAs and PCGs in two cohorts. **(B)** Proportion of nuclear (negative) and cytoplasmic (positive) lncRNAs in two cohorts **(C)** The top 10 differentially expressed TFs associated with EMT-related lncRNAs of two cohorts. **(D)** 19 of 58 identified EMT-related lncRNA with close relations to differentially expressed TFs **(E)** Activated and suppressed TFs by comparing C2 with C1. **(F)** GSEA of genes targeted by 7 upregulated TFs. Size indicates gene counts. **(G)** Comparison of expression of 7 upregulated TFs between C1 and C2 subtypes in TCGA-COAD cohort. Student *t* test was conducted. ****p < 0.0001.

Given that we have illustrated distinct molecular features of different subtypes, we implicated that EMT-related lncRNAs were greatly involved in modulating oncogenic pathways or the expression of immune-related genes. Actually, close associations (both negative and positive) were observed between EMT-related lncRNAs and protein-coding genes (PCGs) (Figure 7A). It is known that the function of lncRNAs is highly associated with their subcellular locations. Therefore, to evaluate the possible mechanism of the regulation, we assessed the locations of these 58 identified EMT-related lncRNAs. We found over a half of lncRNAs localized in the nuclear with 61.59% and 63.30% in TCGA-COAD and GSE17538 cohorts respectively (Figure 7B). As most of EMT-related lncRNAs localized in the nuclear, we supposed that they possibly regulated gene expression by interacting with TFs. We then analyzed the TF activity and screened differentially expressed TFs between two subtypes (131 TFs in TCGA-COAD and 106 TFs in GSE17538). Correlation analysis between 58 EMT-related lncRNAs and dysregulated TFs discovered a group of important TFs and lncRNAs that may closely interact with each other. The top 10 identified TFs in two cohorts were listed (Figure 7C), and 19 EMT-related lncRNAs were screened to have a close relation to differentially expressed TFs (Figure 7D). By comparing C2 with C1 subtype, we found that a majority of TFs were upregulated in C2 with 7 same TFs upregulated in both two cohorts (Figure 7E). Functional analysis on the genes targeting by these 7 upregulated TFs showed that tumor-related pathways of Jak-STAT signaling and transcriptional misregulation in cancer were significantly enriched (Figures 7F,G). The above results indicated that the 19 identified EMT-related lncRNAs may regulate oncogenic pathways through interacting with the 7 upregulated TFs.

### Six epithelial-mesenchymal transition-related lncRNAs were identified to serve as prognostic biomarkers

**FIGURE 8:**
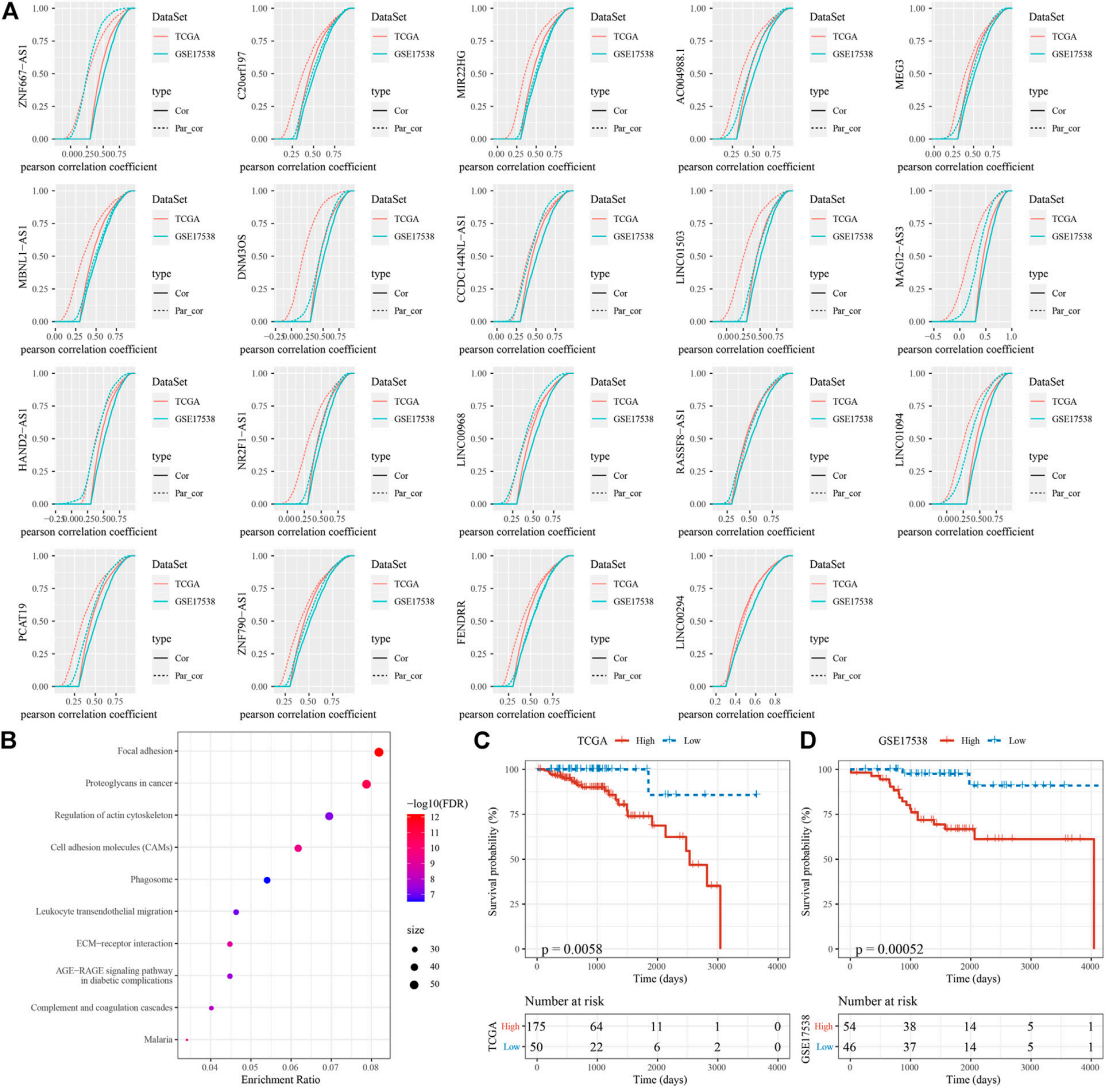
Identification of key EMT-related lncRNAs **(A)** First order partial correlation analysis on the 19 identified EMT-related lncRNAs with EMT score and EMT-related genes. Four CDF curves were presented. Solid and dashed lines represents CDF of correlation coefficients between EMT score and EMT-related genes, without and with adjustment respectively. Kolmogorov-Smirnov test was performed between solid and dashed lines. Vertical axis indicates cumulative probabilities. **(B)** GSEA on EMT-related genes associated with the 6 EMT-related lncRNAs. **(C–D)** Kaplan-Meier survival analysis of high-risk and low-risk groups stratified by the 6-gene prognostic model in two cohorts. Log-rank test was conducted.

In the previous section, we identified 19 EMT-related lncRNAs with a relation to TFs. To understand which lncRNAs among them acted a key role between EMT-related genes and EMT activity, we applied first order partial correlation analysis on them. When eliminating some of lncRNAs, the correlation between EMT activity and EMT-related genes greatly decreased. As a result, six EMT-related lncRNAs were identified, including ZNF667-AS1, CCDC144NL-AS1, MAGI2-AS3, HAND2-AS1, LINC01094 and PCAT19 (Figure 8A). Not surprisingly, GSEA on EMT-related genes associated with these 6 lncRNAs dug out that tumor- and immune-related pathways including proteoglycans in cancer, leukocyte transendothelial migration and focal adhesion were significantly enriched (Figure 8B). Finally, based on the expression of the 6 lncRNAs, we constructed a prognostic model. Samples in two cohorts could be both clearly stratified into high-risk and low-risk groups (*p* = 0.0058 and *p* = 0.00052 in TCGA-COAD and GSE17538 respectively, Figures 8C,D), implicating robust performance of the prognostic model.

## Discussion

A number of studies have found the regulatory role of lncRNAs in EMT and thus promotes tumor development and metastasis. Usually, the function of lncRNAs can be divided into two classifications, EMT promoters or suppressors (Cheng et al., 2019). However, some of lncRNAs are controversial that they act different roles varied by cancer types, indicating the complication of tumor development. To further understand the role of lncRNAs in EMT in colon cancer with early stages (Ⅰ and Ⅱ), we identified 58 lncRNAs that were possibly involved in EMT process, and constructed two molecular subtypes based on these EMT-related lncRNAs.

In the comparison between two subtypes, it was reasonable that C1 subtype had more favorable OS, with lower EMT activity than C2. However, two subtypes displayed no obvious difference on genomic features. Notably, pathways activated in cancer development were observed to be more enriched in C2 subtype, indicating higher invasive activity of tumor cells leading to migration in C2 subtype. There was no doubt in the results that EMT pathway was the most enriched in C2 subtype compared with other enriched tumor-related pathways such as angiogenesis, hypoxia, TNF-α signaling and TGF-β signaling. These pathways were all reported to be associated with EMT process under the regulation of lncRNAs or miRNAs.

Vascular endothelial growth factor (VEGF) contributing for angiogenesis is demonstrated to be linked with Twist2 expression and the reduction of E-cadherin levels (Rojas-Puentes et al., 2016). It has been reported that VEGF upregulation in solid tumors with hypoxia can stimulate the transformation of endothelial cells to mesenchymal cells (Holderfield and Hughes, 2008). Multiple studies on different cancer types demonstrated that VEGF administration induced the detection of EMT markers (Yang et al., 2006; Gonzalez-Moreno et al., 2010; Desai et al., 2013). Hypoxia is a critical hallmark of solid tumors, and are illustrated to induce EMT through hypoxia-inducible factor-1α (HIF-1α) activating SNAI1 (Zhang et al., 2013; Tam et al., 2020). Notably, HIF-1α is a mediator of E-cadherin expression, a major epithelial tumor suppressor, whose reduction can promote angiogenesis (Evans et al., 2007). Links among hypoxia, HIF-1α, E-cadherin, angiogenesis and EMT are shown to promote tumor invasion.

Techasen et alhave demonstrated that tumor necrosis factor-α (TNF-α), an inflammatory cytokine largely secreted from tumor stromal cells, stimulates EMT activation and significantly upregulated Snail expression in cholangiocarcinoma tissues (Techasen et al., 2012). Another critical cytokine tumor growth factor-β (TGF-β) expressing by tumor-infiltrating immune cells also serves as an inducer of EMT through forming EMT-permissive microenvironment, which creates a linkage between EMT and TME (Fuxe and Karlsson, 2012). Moreover, we found that JAK-STAT signaling controlling immune response was highly enriched in C2 subtype. JAK-STAT signaling mediates immune cells in response to cytokines and growth factors, which is involved in metastasis and EMT (Jin, 2020). Xue et alhave revealed that lncRNA-AB073614 promotes EMT through JAK-STAT3 signaling pathway in colorectal cancer cells (Xue et al., 2018). These associations support the reliability of EMT-related lncRNAs for classifying COAD patients into two subtypes where EMT-related pathways were highly enriched in C2 subtype.

Besides enriched pathways, two subtypes also showed distinct TME with high immune infiltration in C2 subtype. A series of cytokines, chemokines and immune checkpoints secreted by tumor cells and tumor-infiltrated immune cells contribute EMT-permissive TME (Jung et al., 2015). Although C2 subtype was highly infiltrated with higher proportion of cytotoxic immune cells, immunosuppressive cells such as macrophages, regulatory T cells, and MDSCs were also highly enriched. In addition, higher expression of immune checkpoints was shown in C2, further supporting the close relation between TME and EMT. In lung adenocarcinoma, Lou et alfound that multiple immune checkpoints associating with increased regulatory T cells including PD-L1, PD-1, TIM-3, B7-H3, BTLA and CTLA-4 displayed EMT phenotype (Lou et al., 2016). Similarly, C2 subtype with high EMT activity also shown increased expression of BTLA, CD274 (PD-L1) and CTLA-4, suggesting that these immune checkpoints may be involved in EMT process.

To understand the possible mechanism of EMT-related lncRNAs regulating EMT-related genes, we investigated the relation between lncRNAs and TFs. We discovered that 7 TFs were significantly upregulated in C2 subtype, and JAK-STAT signaling pathway was found to be enriched in these TFs, which was consistent with the previous findings. Among the 19 identified lncRNAs correlated with TF expression, MEG3 and MIR22HG were massively expressed in the nuclear. MEG3, considering as an oncogenic lncRNA (Al-Rugeebah et al., 2019), is indicated to affect EMT in various cancer types such as breast cancer (Zhang et al., 2017), ovarian cancer (Wang et al., 2019), lung cancer (Terashima et al., 2017) and gastric cancer (Xu et al., 2018). However, it has not been reported in colon cancer, which may serve as a novel direction for further characterizing the mechanism of EMT. MIR22HG is demonstrated as a tumor suppressor through TGF-β/SMAD signaling in colorectal cancer, whose depletion can promote EMT process and tumor metastasis (Xu et al., 2020). We speculated that these 19 lncRNAs are highly involved in regulating EMT through interacting with TFs or other regulators.

Furthermore, to identify key EMT-related lncRNAs, we used first-order partial correlation analysis and dug out six key lncRNAs including ZNF667-AS1, CCDC144NL-AS1, MAGI2-AS3, HAND2-AS1, LINC01094, and PCAT19. These six lncRNAs were all reported to be involved in cancer progression. ZNF667-AS1 was reported to be involved in cancer progression and migration in laryngeal squamous cell carcinoma and cervical cancer (Li et al., 2019; Meng et al., 2019). CCDC144NL-AS1 was identified as a prognostic biomarker in non-small cell lung cancer and it could also promote hepatocellular carcinoma development (Zhang et al., 2021a; Zhang et al., 2021b). MAGI2-AS3 and HAND2-AS1 were dysregulated in many cancer types and were suggested as potential biomarkers for cancer prognosis (Gu et al., 2021; Kai-Xin et al., 2021). LINC01094 could promote the progression of ovarian cancer (Chen et al., 2021), breast cancer (Wu et al., 2021), pancreatic cancer (Luo et al., 2021), and other cancers. PCAT19 could activate cell-cycle genes thereby promoting cancer cell growth and cancer metastasis in pancreatic cancer (Hua et al., 2018).

In conclusion, this study proposed two novel molecular subtypes based on EMT-related lncRNAs. The distinct features of enriched pathways and TME between two subtypes supported that EMT-related lncRNAs played important roles in EMT process through regulating TFs involved in JAK-STAT signaling. Moreover, we identified six key EMT-related lncRNAs associated overall survival and the six lncRNAs could serve as a prognostic signature for COAD patients. As the study focused on COAD samples with stage Ⅰ and Ⅱ, we expanded the fundamental research on the early stages of COAD and the six lncRNAs may serve as biomarkers for early diagnose of COAD. However, the limitation was that only pure bioinformatics analysis was applied in the present study. In addition, we did not distinguish left-sided and right-sided colon cancer, which may lower the accuracy of our results. In the future work, the role and prognostic value of six key EMT-related lncRNAs needed to be further explored and validated in more clinical patients.

## Data Availability

The datasets presented in this study can be found in online repositories. The names of the repository/repositories and accession number(s) can be found in the article/Supplementary Material.
